# Keratin 80 promotes migration and invasion of colorectal carcinoma by interacting with PRKDC via activating the AKT pathway

**DOI:** 10.1038/s41419-018-1030-y

**Published:** 2018-09-27

**Authors:** Changcan Li, Xisheng Liu, Yuan Liu, Xueni Liu, Rangrang Wang, Jianhua Liao, Shaohan Wu, Junwei Fan, Zhihai Peng, Bin Li, Zhaowen Wang

**Affiliations:** 1 Department of General Surgery, Tongji Hospital, Tongji University School of Medicine, Shanghai, 200065 China; 2 Department of General Surgery, Shanghai General Hospital, Shanghai Jiao Tong University School of Medicine, Shanghai, 200080 China; 3 Department of General Surgery, Zhejiang Hospital, Hangzhou, 310013 China; 4 Department of General Surgery, The Second Affiliated Hospital of Jiaxing College, Jiaxing, 314000 China; 5 Shanghai Institute of Immunology, Shanghai Jiao Tong University School of Medicine, Shanghai, 200025 China

## Abstract

Little is known about the function of Keratin 80 (KRT80), an epithelial keratin, in cancer. This study investigated the role of KRT80 in the prognosis of colorectal carcinoma (CRC) and the underlying mechanisms involved in CRC migration and invasion. We analyzed the expression of KRT80 using The Cancer Genome Atlas and Oncomine databases. Higher expression of KRT80 was found to be significantly associated with multiple pathological parameters, lower disease-free survival, and overall survival in CRC patients. Also, KRT80 was an independent prognostic indicator for CRC. Furthermore, altered KRT80 expression impacted migration and invasion of CRC cells, as well as the expression of epithelial–mesenchymal transition (EMT)-related markers and cell morphology via the AKT pathway. Inhibiting the expression of AKT could reverse these phenomena. Liquid Chromatograph Mass Spectrometer/Mass Spectromete, Co-immunoprecipitation, and laser scanning confocal microscopy techniques showed that KRT80 could interact with protein kinase, DNA-activated, catalytic polypeptide (PRKDC). Suppressing PRKDC could inhibit the expression of AKT and EMT, as well as the migration and invasion of CRC cells. Taken together, these results demonstrated that KRT80 was an independent prognostic biomarker for CRC and promoted CRC migration and invasion by interacting with PRKDC via activation of the AKT pathway.

## Introduction

Although prominent advances have been made in the treatment of colorectal carcinoma (CRC), it remains the most common gastrointestinal cancer worldwide, with high morbidity and mortality rates^1^. Around 25% of newly diagnosed patients with CRC have metastasis, whereas ~ 50% of patients with localized tumors will eventually develop metastasis, with most of these tumors being unresectable^2^. The available clinical biomarkers have unsatisfactory sensitivity and specificity for CRC prognostic evaluation^3^. Identifying specific altered genes and/or biomarkers with high sensitivity and specificity will be invaluable for early diagnosis and prognosis of CRC.

Keratins are intermediate filament cytoskeletal proteins of epithelial cells that are responsible for their structural integrity^4^. Keratins can be divided into two types: acidic or type I and basic or neural type II. Keratin 80 (KRT80) belongs to type II, along with Keratin 7 (KRT7), Keratin 8 (KRT8), and Keratin 78 (KRT78). KRT80 gene is located on chromosome 12q13 and encodes a 452-amino-acid protein with a calculated molecular mass of 50.5 kDa^5,6^. Keratins are expressed in many tumors including carcinomas, sarcomas, and trophoblastic neoplasms^7^. Keratins are tissue-specific and expressed in a differentiation-dependent manner. They are typical markers for epithelial cells^8^. Several studies have reported that keratins are extensively expressed in many malignant epithelial cells and play an important role in the regulation of cell migration and invasion^9,10^. However, the correlation between KRT80 expression and cancer has not been reported, not to mention in CRC. Hence, we focused on the role of KRT80 in CRC.

In this study, the clinical significance and related mechanisms of KRT80 in CRC were investigated. The Cancer Genome Atlas (TCGA) and Oncomine databases were used to predict the expression of KRT80 in CRC. High expression of KRT80 was demonstrated to be an independent prognostic indicator for CRC. Next, in vitro experiments showed that KRT80 was related to migration and invasion of CRC cells via the AKT pathway, the expression of epithelial–mesenchymal transition (EMT) markers and change of CRC cell morphology. Using Liquid Chromatograph Mass Spectrometer/Mass Spectrometer (LC-MS/MS), Co-immunoprecipitation (Co-IP), and laser scanning confocal microscopy (LSCM), we demonstrated that KRT80 interacted with Protein Kinase, DNA-Activated, Catalytic Polypeptide (PRKDC), also called as DNA-Dependent Protein Kinase Catalytic Subunit in CRC. Suppression of PRKDC could inhibit the AKT pathway, EMT, and the migration and invasion of CRC cells. These findings showed that KRT80 may play a vital role in CRC.

## Results

### KRT80 is upregulated in human CRC tissues

**Fig. 1:**
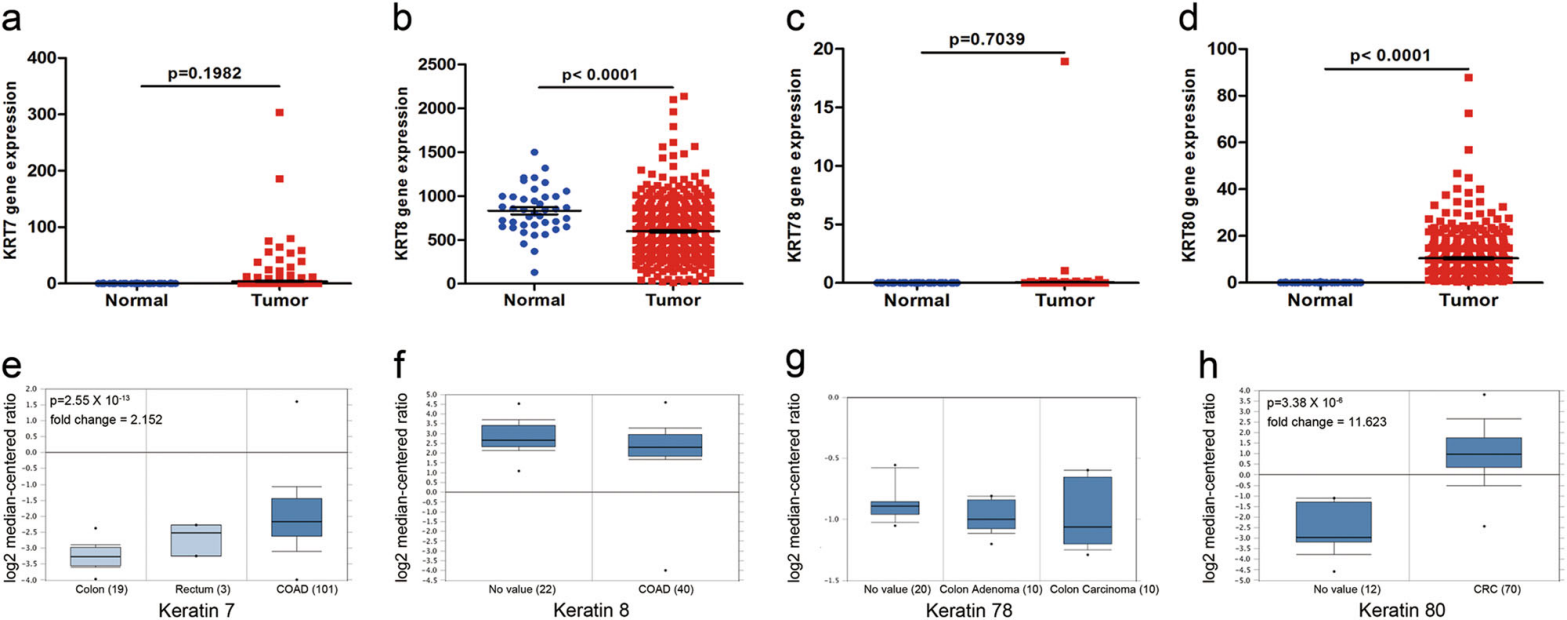
Keratin family type II RNA expression on The Cancer Genome Atlas (TCGA) and the Oncomine databases **a–d** TCGA showed expression of keratin 7 (KRT7), keratin 8 (KRT8), keratin 78 (KRT78), and keratin 80 (KRT80) in normal mucosa and colon adenocarcinoma (COAD). **e** KRT7 expression in colorectum (TCGA Colorectal). **f** KRT8 expression in colon (Alon Colon). **g** KRT78 expression in colon (Skrzypczak Colorectal 2). **h** KRT80 expression in colorectum (Skrzypczak Colorectal 2)

The keratin family type II RNA expression was examined in the TCGA and Oncomine databases. Only KRT80 showed significant changes in CRC tissues, as compared with normal adjacent mucosa (Fig. 1d, h). KRT7, KRT8, and KRT78 had no significant changes (Fig. 1a–c, e–g).

**Fig. 2:**
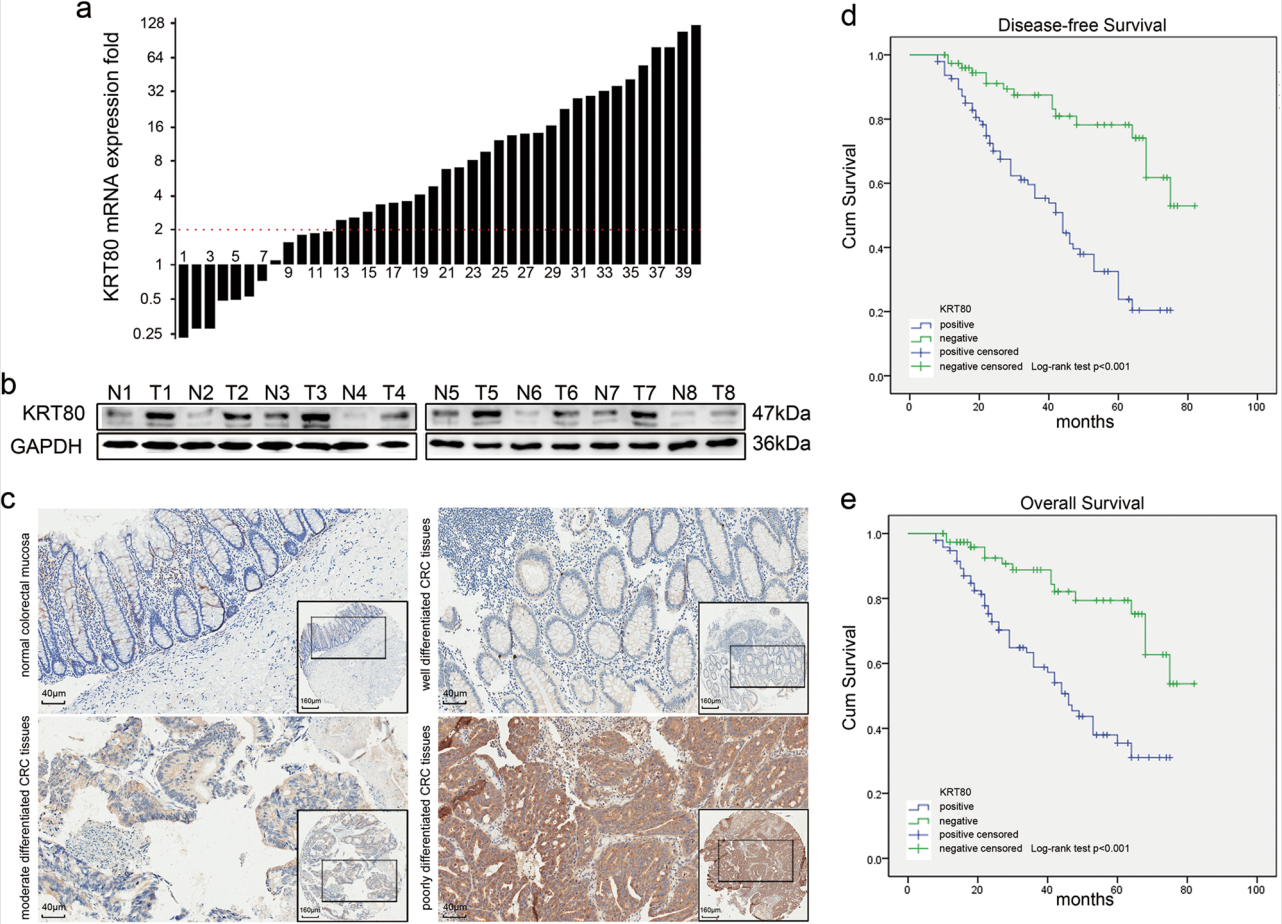
KRT80 expression in colorectal tissues and its value in prognosis **a** KRT80 mRNA expression in 40 tumor tissues and paired adjacent normal mucosa by quantitative real-time polymerase chain reaction (qRT-PCR). The logarithmic scale of 2^-ΔΔCt^ was used to measure the fold-change. β-actin as internal reference. **b** Western blot was performed to check KRT80 protein expression in eight paired colorectal carcinoma (CRC) tissue samples, with GAPDH as loading control. N, normal tissue; T, tumor tissue. **c** Representative immunohistochemical (IHC) staining of KRT80 in normal colorectal mucosa and cancerous tissues. **d**, **e** Kaplan–Meier analysis showed disease-free survival (DFS) and overall survival (OS) of CRC patients with KRT80 expression

Next, 40 paired CRC samples were assessed for KRT80 mRNA expression by quantitative real-time PCR (qRT-PCR). The relative expression (ΔCt) of KRT80 in cancerous tissues was 10.56 ± 1.60, and 13.07 ± 2.29 in adjacent normal tissues (*p* *<* 0.001). A total of 33 tumor samples (82.5%) showed higher KRT80 expression in CRC tissues as compared with paired normal mucosa, with 28 tumor samples (70%) having at least a twofold increase (Fig. 2a). Western blot analysis confirmed that the protein level of KRT80 was significantly upregulated in CRC tissue samples as compared with matched normal tissues (Fig. 2b). These results demonstrated that KRT80 expression was increased at the transcriptional and translational levels in CRC tissues.

### Association between KRT80 expression and clinicopathological parameters for CRC

To determine the association of clinicopathological characteristics with KRT80 expression, immunohistochemistry (IHC) analysis was performed using a tissue microarray (TMA) containing 120 primary CRC samples and paired adjacent normal mucosae. KRT80 localized in the cytoplasm, and significant differences were observed in KRT80 expression between normal mucosa and CRC tissues (Fig. 2c). KRT80 protein staining was higher in the primary cancer samples as compared with adjacent normal mucosa (Table 1). Also, KRT80 staining increased with the increase in tumor grade. Elevated KRT80 expression was significantly associated with several clinicopathological factors, including T stage, N stage, M stage, the American Joint Committee on Cancer (AJCC) stage, differentiation, and vessel invasion (Table 2).

**Table 1:** Expression of KRT80 protein in paired normal tissues and CRC tissues

Tissue sample	*n*	KRT80 expression		*P* value
		Negative (%)	Positive (%)	
Normal tissue	120	65(54.2)	48(45.8)	0.01^a^
Cancerous tissue	120	48(40.0)	72(60.0)	

^a^ Implies statistical difference, *P* value is based on the chi-square test

**Table 2:** Correlation between KRT80 expression and clinicopathological features

Parameters	Total (*n* = 120)	KRT80 protein expression		*P* value
		Negative (*n* = 55)(%)	Positive (*n* = 65)(%)	
Age, years				
< 65	53	26(47.3)	27(41.5)	0.387
≥ 65	67	29(52.7)	38(58.5)	
Gender				
Male	67	32(58.2)	35(53.8)	0.577
Female	53	23(41.8)	30(46.2)	
Location				
Ascending	19	8(14.5)	11(16.9)	0.958
Transverse	8	3(5.5)	5(7.7)	
Descending	14	7(12.7)	7(10.8)	
Sigmoid	43	21(38.2)	22(33.8)	
Rectum	36	16(29.1)	20(30.8)	
T stage				
T1 + T2	58	34(61.8)	24(36.9)	0.013^a^
T3 + T4	62	21(38.2)	41(63.1)	
N stage				
N0	86	47(85.5)	39(60.0)	< 0.001^a^
N1	34	8(14.5)	26(40.0)	
M stage				
M0	107	52(94.5)	55(84.6)	0.004^a^
M1	13	3(5.5)	10(15.4)	
AJCC stage				
I + II	59	36(65.5)	23(35.4)	0.001^a^
III + IV	61	19(34.5)	42(64.6)	
Differentiation				
Well	47	30(54.5)	17(26.2)	0.006^a^
Moderate	49	19(34.6)	30(46.1)	
Poor	24	6(10.9)	18(27.7)	
Vessel invasion				
No	101	51(92.7)	50(76.9)	0.006^a^
Yes	19	4(7.3)	15(23.1)	

^a^*P* < 0.05 indicates a significant association among the variables

### Survival analysis and prognostic significance of KRT80 expression in CRC

To assess the association between KRT80 expression and survival of CRC patients, Kaplan–Meier curves with log-rank test was used to determine disease-free survival (DFS) and overall survival (OS) in 120 CRC patients. The group with high levels of KRT80 expression in tumors had poorer DFS and OS as compared with the group with lower KRT80 expression (Fig. 2d, e).

In addition, Cox proportional hazards model was used for univariate and multivariate analyses of DFS and OS. In the univariate analysis, T stage, N stage, M stage, AJCC stage, differentiation, vessel invasion, and KRT80 expression were associated with DFS and OS (Table 3). To further investigate the relationship between the patients’ prognosis and individual parameters, multivariate analysis was performed for all significant factors derived from the univariate analysis. The results showed that M stage, AJCC stage and KRT80 expression were independent prognostic factors for DFS and OS (Table 4).

**Table 3:** Univariate analysis for disease-free survival (DFS) and overall survival (OS)

Variable	DFS			OS		
	HR	95% CI	*P* value	HR	95% CI	*P* value
Age, years						
< 65	—			—		
≥ 65	0.962	0.605–1.530	0.870	1.071	0.648–1.772	0.788
Gender						
Male	—			—		
Female	0.872	0.543–1.399	0.570	1.140	0.685–1.897	0.614
Location						
Ascending	—			—		
Transverse	1.297	0.622–2.705	0.487	1.438	0.655–3.158	0.365
Descending	1.978	0.786–4.979	0.148	2.340	0.909–6.022	0.078
Sigmoid	1.301	0.570–2.973	0.532	0.730	0.244–2.186	0.574
Rectum	1.112	0.627–1.973	0.717	1.284	0.695–2.372	0.424
T stage						
T1+T2	—			—		
T3+T4	2.057	1.251–3.381	0.004^a^	2.200	1.281–3.777	0.004^a^
N stage						
N0	—			—		
N1	2.154	1.353–3.429	0.001^a^	2.474	1.497–4.087	< 0.001^a^
M stage						
M0	—			—		
M1	3.262	1.891–5.629	< 0.001^a^	4.000	2.281–7.014	< 0.001^a^
AJCC stage						
I + II	—			—		
III + IV	2.451	1.519–3.956	< 0.001^a^	2.580	1.536–4.334	< 0.001^a^
Differentiation						
Well	—			—		
Moderate	2.278	1.238–4.192	0.008^a^	2.486	1.274–4.852	0.008^a^
Poor	3.560	1.865–6.797	< 0.001^a^	3.700	1.833–7.468	< 0.001^a^
Vessel invasion						
No	—			—		
Yes	2.067	1.197–3.568	0.009^a^	2.166	1.208–3.884	0.010^a^
KRT80 expression						
Negative	—			—		
Positive	3.897	2.213–6.864	< 0.001^a^	3.397	1.882–6.132	< 0.001^a^

HR hazard ratio, *CI* confidence interval

^a^*P* < 0.05 was considered significant

**Table 4:** Multivariate analysis for DFS and OS

Variable	DFS			OS		
	HR	95% CI	*P* value	HR	95% CI	*P* value
T stage	1.249	0.729–2.140	0.419	1.513	0.832–2.750	0.175
N stage	1.184	0.709–1.979	0.518	1.301	0.743–2.279	0.358
M stage	2.675	1.104–6.481	0.029^a^	4.364	1.641–11.603	0.003^a^
AJCC stage	2.192	1.297–3.704	0.003^a^	2.154	1.222–3.796	0.008^a^
Differentiation						
Well	—					
Moderate	1.920	0.997–3.698	0.051	2.122	1.039–4.337	0.039^a^
Poor	2.115	1.048–4.267	0.037^a^	1.978	0.924–4.232	0.079
Vessel invasion	0.869	0.373–2.022	0.744	0.609	0.232–1.598	0.313
KRT80 expression	2.527	1.390–4.594	0.002^a^	2.023	1.085–3.771	0.027^a^

*HR* hazard ratio, *CI* confidence interval

^a^*P* *<* 0.05 was considered significant

### Construction of stable CRC cells with KRT80 knockdown or overexpression

**Fig. 3:**
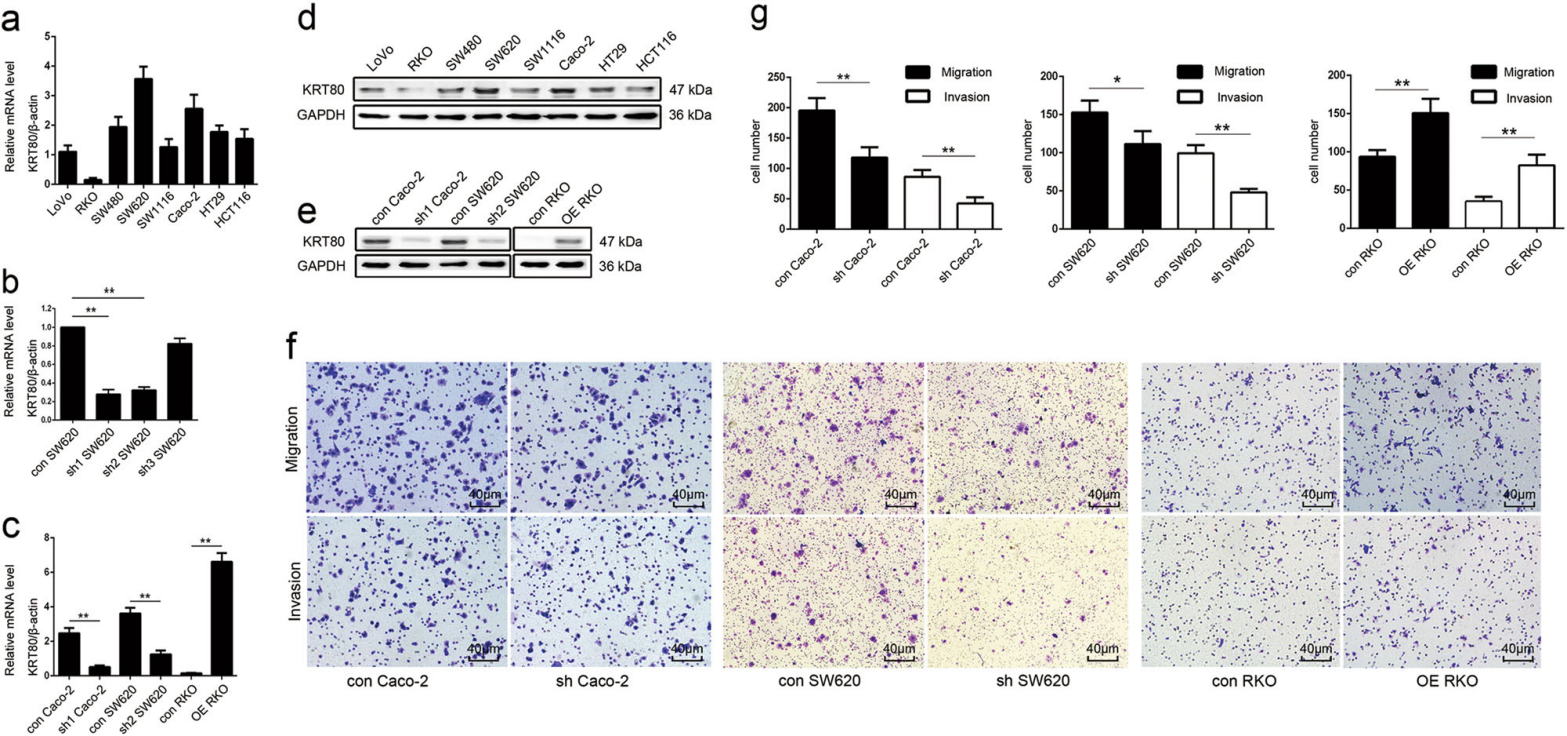
The expression of KRT80 and its function in migration and invasion of CRC cells **a**, **d** The expression of KRT80 mRNA and protein in CRC cells. **b**, **c**, **e** KRT80 mRNA and protein expression in CRC cells with KRT80 downregulation or overexpression. **f**, **g** The effect of KRT80 on migration or invasion. (*P < 0.05, **P < 0.01)

**Fig. 4:**
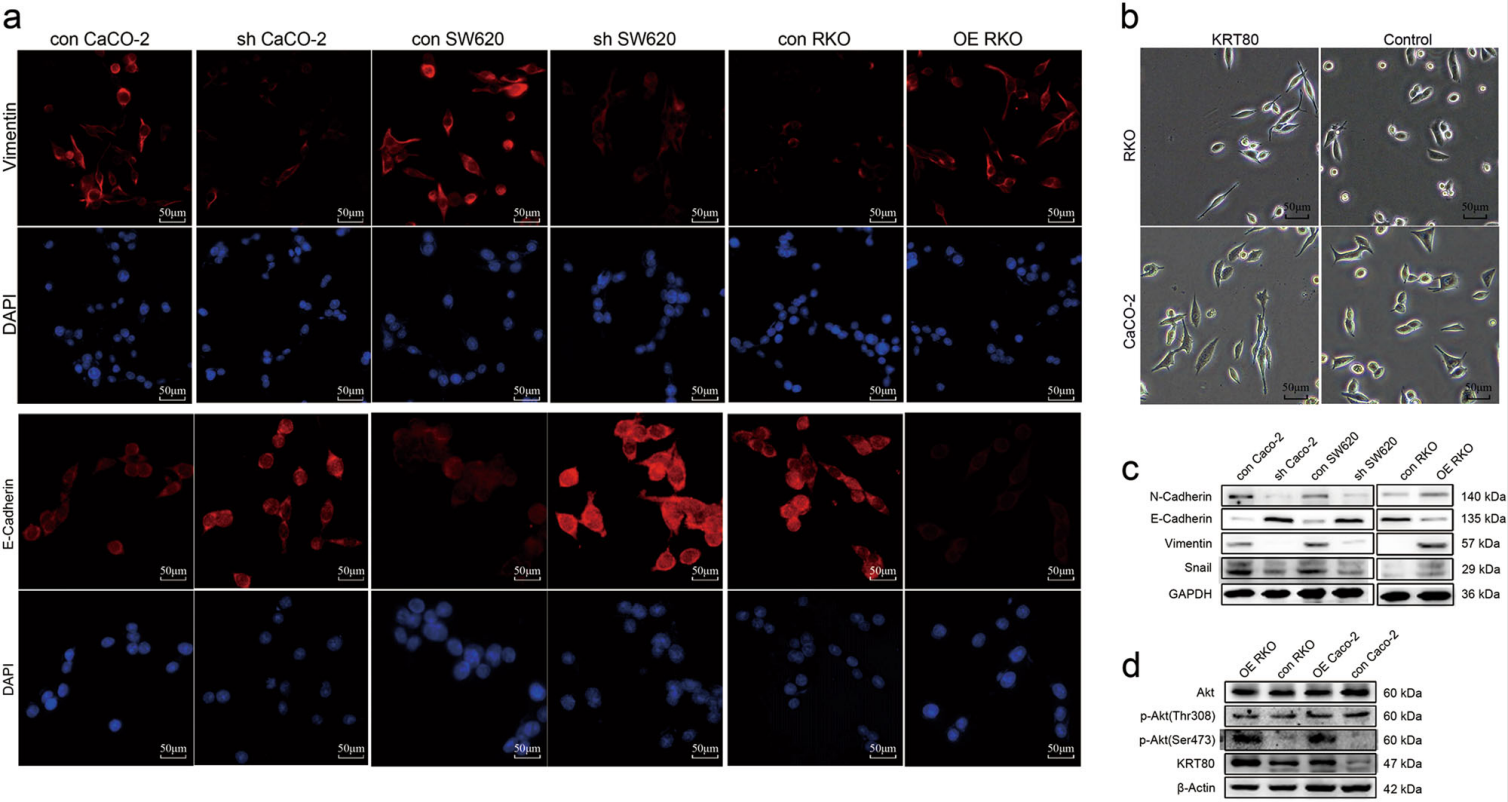
KRT80 regulated change in cell morphology and expression of epithelial–mesenchymal transition (EMT)-related markers via AKT pathway in CRC cells **a** Immunofluorescence (IF) staining was used to examine the expression of Vimentin and E-Cadherin. **b** Cell morphology change in CRC cells transferred with KRT80 or blank vector. **c** Western blot analysis showed changing of EMT markers in CRC cells. **d** Western blot showed the expression of AKT in CRC cells

First, we checked the expression level of KRT80 in different CRC cells. The results showed that KRT80 was highly expressed in SW620 and Caco-2 cells and lower in RKO cells, as compared with other cells (Fig. 3a, d). To evaluate efficiency of different shRNA sequences and avoid off-target effects, we constructed three plasmids with different shRNA sequences and individually transfected them into SW620 cells. The efficiencies of the shRNAs were confirmed by qRT-PCR (Fig. 4b). Then, shRNA1 and shRNA2 were individually transfected into Caco-2 and SW620 cells. KRT80 plasmid was transfected into RKO cells. Knockdown or overexpression of KRT80 was confirmed by qRT-PCR and western blot (Fig. 3c, e).

### KRT80 promotes CRC cell migration and invasion, changes in cell morphology, and EMT markers via the AKT pathway

Cell migration and invasion are necessary for tumor development and metastasis^11^. We performed transwell assay to assess the impact of KRT80 expression on cell migration and invasion. Knockdown of KRT80 suppressed the migration and invasion of CRC cells, whereas overexpression of KRT80 promoted these activities (Fig. 3f, g).

Numerous studies have proven that EMT is necessary for metastasis of malignant cancer^12–14^. Epithelial cells lose their polarity, and acquire migratory and invasive capabilities^15^. The micrographs showed that overexpression of KRT80 in CRC cells can change their morphology from round to polygonal, along with the induction of EMT (Fig. 4b). Immunofluorescent (IF) assay showed lower expression of Vimentin, a mesenchymal marker, after suppression of KRT80, whereas the expression of E-Cadherin, an epithelial marker, was increased (Fig. 4a). Besides, western blot analysis was performed to determine the role of KRT80 in EMT in CRC cells. E-Cadherin was upregulated after KRT80 was suppressed, whereas mesenchymal markers were downregulated, including N-Cadherin, Vimentin, and Snail (Fig. 4c).

The AKT pathway can participate in CRC migration and invasion, so we examined the changes in AKT^16^. Overexpression of KRT80 promoted the expression of p-AKT (Ser 473), whereas the expression of total AKT and p-AKT (Thr 308) showed no significant changes (Fig. 4d).

### Suppression of AKT can inhibit EMT expression, cell migration, and invasion

**Fig. 5:**
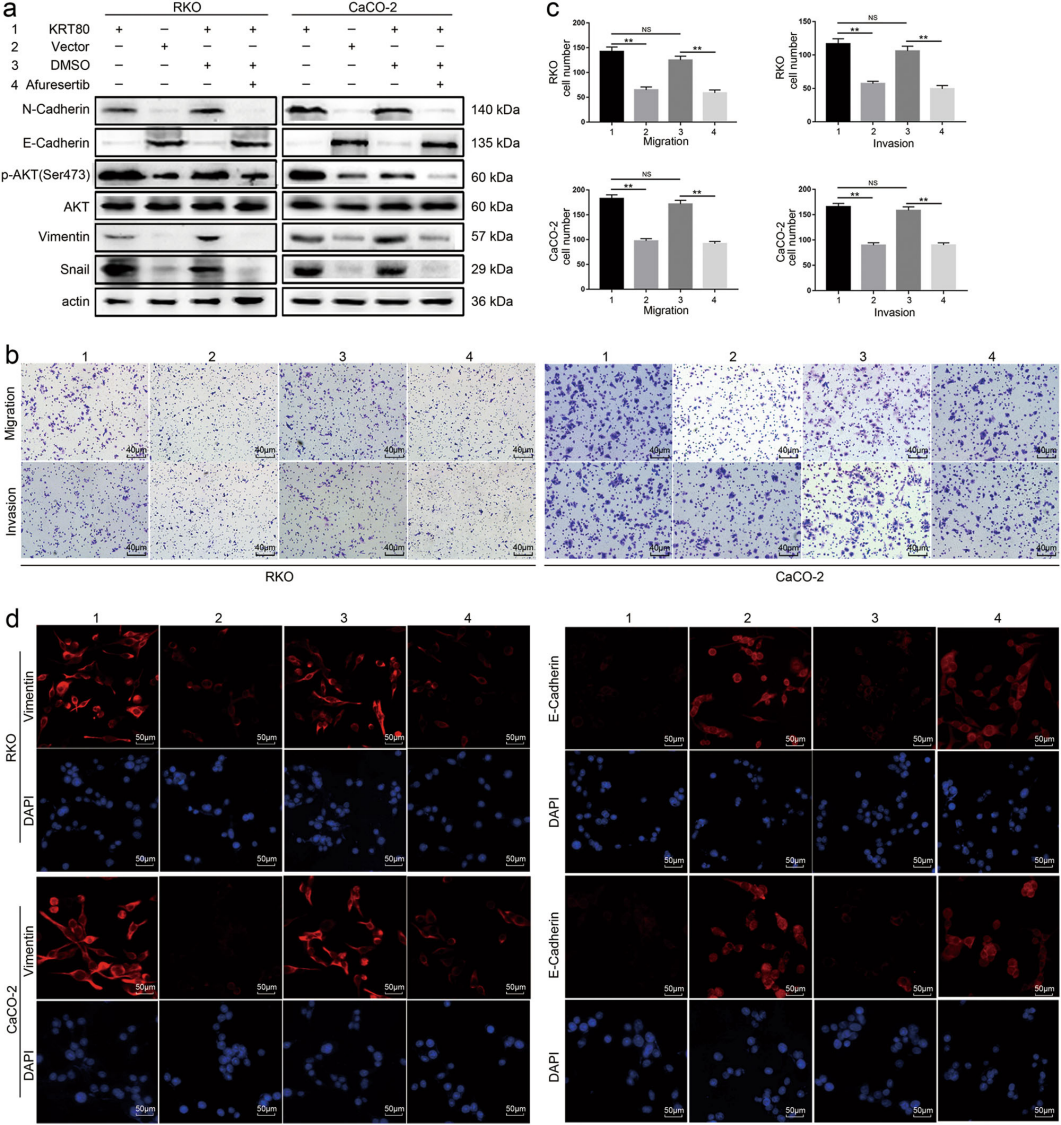
The function variation of CRC cells as AKT was inhibited by Afuresertib **a** Western blot analysis showed AKT inhibitor Afuresertib inhibited the activation of AKT pathway induced by KRT80, and variation of EMT markers in CRC cells. **b**, **c** Afuresertib inhibited migration and invasion of CRC cells. **d** IF staining showed the expression of Vimentin and E-Cadherin after Afuresertib was used

Afuresertib was reported to be a highly efficient AKT pathway inhibitor^17^. In order to check the function of AKT in CRC, we inhibited AKT expression with Afuresertib. The results showed that suppression of AKT could inhibit the expression of mesenchymal markers, as well as cell migration and invasion, whereas the expression of E-Cadherin was upregulated (Fig. 5).

### KRT80 interacts with PRKDC in CRC cells

**Fig. 6:**
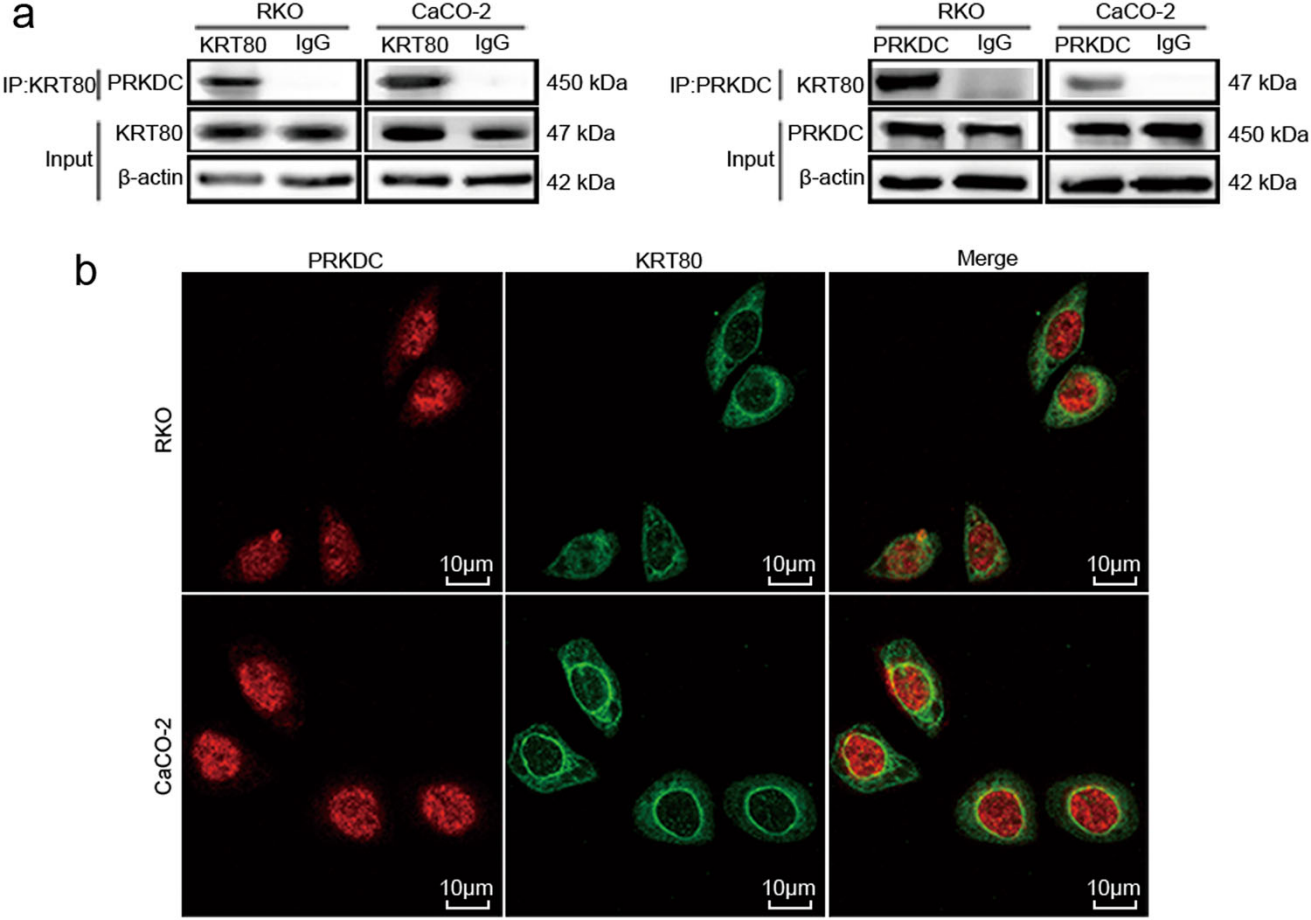
KRT80 can interact with PRKDC in CRC cells **a** Co-Immunoprecipitation (CO-IP) assay showed KRT80 can interact with PRKDC in CRC cells. **b** Laser scanning confocal microscopy (LSCM) technique was used to show the colocalization of KRT80 and PRKDC in CRC cells

PRKDC is a member of the phosphatidylinositol-3-kinase family^18^. Previous reports have shown that PRKDC is important for AKT activation^19,20^. The results of LC-MS/MS showed that KRT80 may interact with PRKDC, so Co-IP assay was performed to test the prediction (Fig. 6a). Next, we used LSCM to assess potential colocalization between KRT80 and PRKDC. The results demonstrated that KRT80 and PRKDC were colocalized mainly around nuclear membranes in CRC cells (Fig. 6b). These data revealed that KRT80 can interact with PRKDC in CRC cells.

### Suppression of PRKDC expression can inhibit the expression of AKT and EMT, cell migration, and invasion

**Fig. 7:**
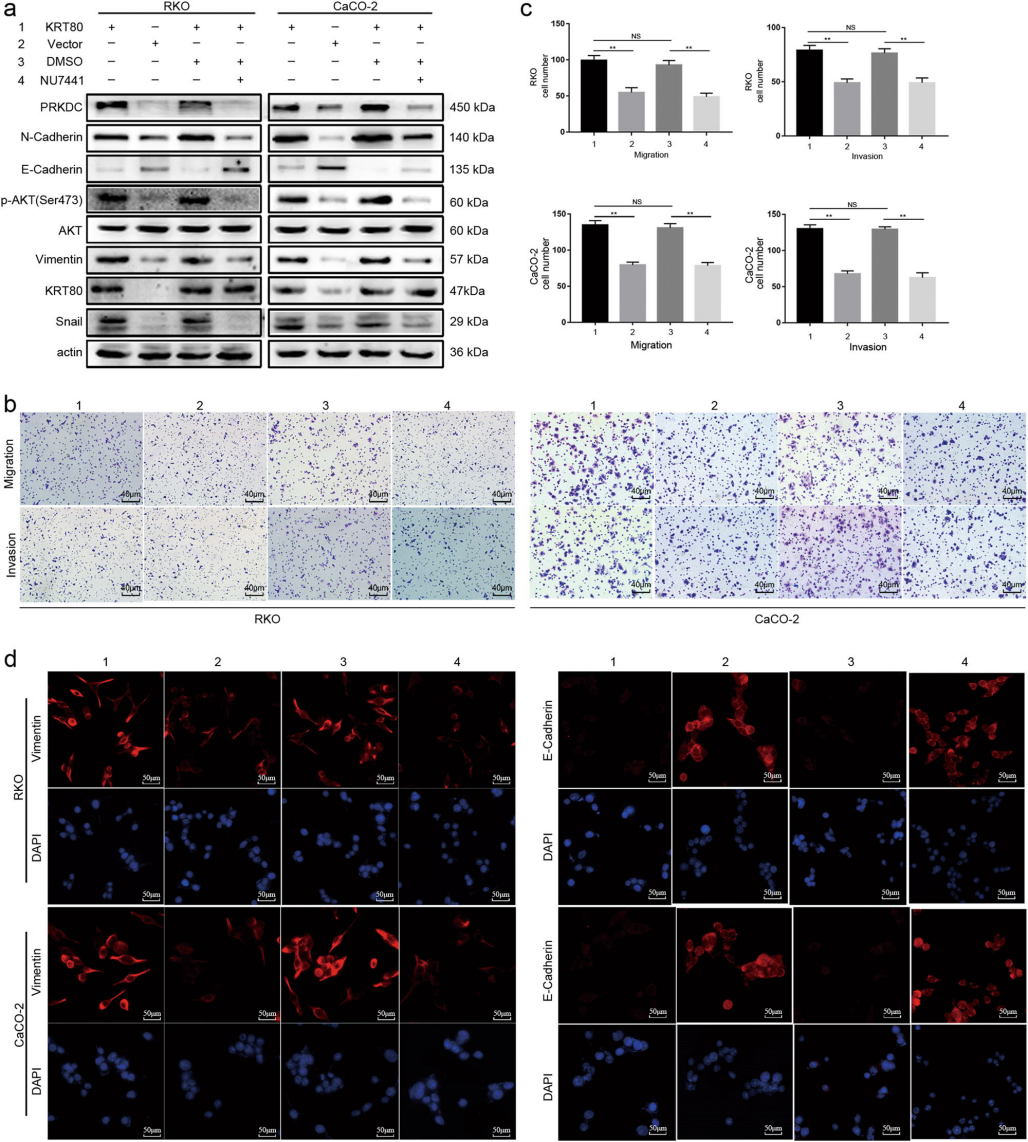
Inhibit PRKDC expression with NU7441 in CRC cells **a** Western blot analysis showed PRKDC inhibitor NU7441 inhibited the expression of PRKDC, which further inhibited the activation of AKT pathway induced by KRT80 and EMT markers in CRC cells. **b**, **c** The suppression of PRKDC with NU7441 inhibited migration and invasion of CRC cells. **d** IF staining showed the expression of Vimentin and E-Cadherin were changed as NU7441 inhibited PRKDC expression

NU7441 can inhibit the expression of PRKDC^21^. Thus, we inhibited PRKDC expression with NU7441, which led to inhibition of the expression of AKT and mesenchymal markers as well as CRC cell migration and invasion, whereas the expression of E-Cadherin was upregulated (Fig. 7). Hence, KRT80 can interact with PRKDC, followed by activation of the AKT pathway, which influences cell migration and invasion.

## Discussion

Keratins are markers of epithelial cells^22^. They expressed in normal and malignant cells, and are associated with tumor malignancy and metastasis^23^. Several keratins have been used as prognostic predictors for different carcinomas of epithelial origin^24–26^. Studies have demonstrated that keratins play a vital role in maintaining the mechanical stability and integrity of epithelial cells, and participate in several intracellular signaling pathways involved in cell stress, proliferation, and metastasis^27,28^. Numerous studies have found several genes that were specifically upregulated or downregulated in CRC tissues and could be used as biomarkers for diagnosis or prognosis^25,29^. KRT80 has been rarely reported to be associated with any disease, including cancer.

This study demonstrated that KRT80 was significantly upregulated in CRC, and elevated KRT80 expression correlated with CRC clinicopathological parameters. Patients with KRT80-positive tumor staining had shorter DFS and OS. In addition, Cox regression models showed that KRT80 was an independent prognostic marker for CRC. Furthermore, KRT80 could promote cell migration and invasion. Besides, overexpression of KRT80 could change CRC cell morphology, from round to polygonal. Moreover, the expression of mesenchymal markers were upregulated, whereas epithelial markers were downregulated because of the close relationship between EMT and metastasis of malignant tumors^13^.

Taken together, these results confirmed that KRT80 can promote CRC migration and invasion. Though majority of the previous studies on AKT focused on its function in proliferation, some studies reported its function in metastasis^30,31^. This study was focused on metastasis and revealed that the AKT pathway was strongly associated with CRC migration and invasion since the AKT inhibitor Afuresertib could inhibit these processes.

PRKDC is involved in the ligation step of the non-homologous end joining pathway of DNA double strand break repair. It also participates in modulation of transcription, maintaining telomere length, regulation of apoptosis, regulation of mitochondrial protein function, and phosphorylation of numerous proteins^32–34^. Also, as a biomarker in malignant tumors, PRKDC participates in tumor metastasis in prostate cancer, laryngeal squamous cell carcinoma and other malignant tumors^35–37^. PRKDC plays numerous functions in tumors, and hence we used LC-MS/MS to examine its function in this study. The result showed that PRKDC can interact with KRT80, and can influence AKT and cell function.

This is the first report on the function of KRT80 in malignant tumors. KRT80 expression was upregulated in CRC samples, which significantly correlated with poor survival. These results suggested that KRT80 may be a novel biomarker for the diagnosis of CRC and a therapeutic target for CRC.

## Materials and methods

### Databases analysis

With the approval of the project by the consortium, TCGA RNA-Seq were downloaded from TCGA website (https://cancergenome.nih.gov/). Oncomine (https://www.oncomine.org/) was used to acquire KRT80 RNA expression in CRC and normal mucosae.

### Patients and tissue samples

One hundred and twenty CRC tissue samples for TMA construction and other 40 CRC tissues were obtained from the Department of General Surgery, Shanghai General Hospital. Patients who had received chemotherapy or radiotherapy were excluded from this study. DFS and OS was defined as the interval between the initial surgery to clinical or radiological determined recurrence/metastasis or death. Tumor staging was based on pathological outcomes based on the guidelines of AJCC. The diagnoses were confirmed by at least two clinical pathologists. Written informed consents were obtained from all patients. The study was approved by the Ethics Committee of Shanghai General Hospital.

### QRT-PCR

Total RNA was isolated from 40 pairs of CRC tissues and cell lines, then was used to synthesize cDNA (TaKaRa, Tokyo, Japan), according to the manufacturer’s instructions. KRT80 mRNA levels were quantitated using SYBR Green Master Mix Kit (Takara), qRT-PCR was performed using the Real-time PCR System (Applied Biosystems, Foster City, CA, USA). The amplification conditions used were as follows: initial denaturation for 30 s at 95 °C, 40 cycles of denaturation for 5 s at 95 °C, annealing for 30 s at 60 °C, and elongation for 30 s at 72 °C, with a final extension step for 30 s at 72 °C. RT-qPCR was performed in triplicate and the fold-change (2^-ΔΔCt^) of KRT80 expression was calculated for each group. The primers used for quantitative PCR are:

KRT80 forward: 5′- GCTGCTCTTGCCATAATCAA-3′

KRT80 reverse: 5′-AATGCTCCTGCCCAATCTC-3′

β-actin forward: 5′-CATGTACGTTGCTATCCAGGC-3′

β-actin reverse: 5′-CTCCTTAATGTCACGCACGAT-3′

### Protein extraction and western blot

Total protein from CRC tissue samples and cells were extracted using radioimmunoprecipitation assay buffer (RIPA) Lysis Buffer (Beyotime Biotechnology Co., Jiangsu, China). Western blot was performed as previously described^38^. The following primary antibodies were used: anti-KRT80 (1:1000, Proteintech Group, Inc., Rosemont, IL, USA), anti- N-Cadherin (1:1000, Cell Signaling Technology, CST, Danvers, MA, USA), anti-E-Cadherin (1:1000, CST), anti-Vimentin (1:1000, CST), anti-Snail (1:1000, CST), anti-AKT (1:1000, CST), anti-p-AKT (Thr 308) (1:1000, CST), anti-p-AKT (Ser 473) (1:1000, CST), anti-PRKDC (1:1000, CST), anti-GAPDH (1:1000, CST), anti-β-Actin (1:1000, CST).

### IHC

TMA that included 120 CRC tissues were constructed by commercial company (Outdo Biotech Co. Shanghai, China). IHC staining of TMA and the calculating method were described before^39^.

### Plasmid transfection

KRT80 shRNA was constructed by Genechem Co. Ltd. (Shanghai, China) and was used to silence KRT80. Genechem (Shanghai) also synthesized the KRT80 plasmid. The KRT80 shRNA and KRT80 primer sequences are listed below:

shRNA1 forward: GCACTATCTCCAAGGTGACTGTGAA

shRNA1 reverse: TTCACAGTCACCTTGGAGATAGTGC

shRNA2 forward: GGATGCAGAGTGTCTTCATCG

shRNA2 reverse: CGATGAAGACACTCTGCATCC

shRNA3 forward: GCCATTGCCTGAAACTGGAGGAGAA

shRNA3 reverse: TTCTCCTCCAGTTTCAGGCAATGGC

KRT80 forward: ATGGCCTGCCGCTCCTGCGTGGTT

KRT80 reverse: TTACTCTGAGACCTCCGACTCCT

CRC cells were infected with 5 × 10^5^ transducing units/ml of lentiviral particles. Stable cell lines were established after antibiotic selection using 1 μg/ml puromycin (Sigma, St. Louis, MO, USA). KRT80 expression was confirmed in CRC cells using qRT-PCR and western blot analysis.

### Migration and invasion assays

In total, 1 × 10^5^ cells cultured in fetal bovine serum (FBS)-free media were seeded onto the upper chambers of transwell (Corning, NY, USA), and media with 10% FBS was added to the lower chamber. Upper chamber coated without Matrigel (Coring) for migration assay or with Matrigel for invasion assay. After incubating for appropriate intervals, chamber was fixed in methanol and then stained using crystal violet (Beyotime). Using a light microscope, at least five randomly selected fields were photographed following which the counts were averaged. All experiments were performed in triplicate.

### IF staining

Cells were seeded into six well plate with 10 mm coverslip for 24 h. After washing with phosphate-buffered saline, cells were fixed in 4% paraformaldehyde. Then incubated with primary antibody overnight at 4 °C. After incubating the specimen with fluorochrome-conjugted secondary antibody and 4′,6-diamidino-2-phenylindole lucifugally. Images were obtained with a fluorescence microscope.

### Co-IP assays

The cells were incubated in weak-potency RIPA lysis buffer (Beyotime) for 30 min at 4 °C, then centrifuged at 12 000 g for 30 min. Antibodies against PRKDC and KRT80 were added with protein A and G agarose beads (Sigma), and incubated at 4 °C for 6 h. After washing, the complexes were boiled and subjected to western blotting analysis.

### Statistical analysis

All data were presented as mean ± SD for continuous variables or frequencies and percentages for categorical data. Comparisons between the two groups were performed using the students’ *t* test and three-group comparisons were performed using one-way analysis of variance. The *χ*^2^ or Fisher’s exact tests was used to determine the significance of difference between KRT80 and clinicopathological variables. Kaplan–Meier analyses with log-rank test were used to evaluate DFS and OS. The Cox proportional hazard model was used to investigate the hazard ratio and 95% confidence intervals for DFS and OS. All data analysis was performed using the SPSS 20.0 software (SPSS Inc, Chicago, IL, USA). *P* value < 0.05 was considered statistically significant.
